# Blood pressure documentation in the emergency department

**DOI:** 10.1590/S1679-45082017AO3737

**Published:** 2017

**Authors:** Ana Carolina Queiroz Godoy Daniel, Juliana Pereira Machado, Eugenia Velludo Veiga

**Affiliations:** 1 Hospital Israelita Albert Einstein, São Paulo, SP, Brazil.; 2 Centro Universitário Barão de Mauá, Ribeirão Preto, SP, Brazil.; 3 Escola de Enfermagem de Ribeirão Preto, Universidade de São Paulo, Ribeirão Preto, SP, Brazil.

**Keywords:** Nursing records, Blood pressure determination, Monitoring, physiologic, Emergency service, hospital

## Abstract

**Objective:**

To analyze the frequency of blood pressure documentation performed by nursing professionals in an emergency department.

**Methods:**

This is a cross-sectional, observational, descriptive, and analytical study, which included medical records of adult patients admitted to the observation ward of an emergency department, between March and May 2014. Data were obtained through a collection instrument divided into three parts: patient identification, triage data, and blood pressure documentation. For statistical analysis, Pearson’s correlation coefficient was used, with a significance level of α<0.05.

**Results:**

One hundred fifty-seven records and 430 blood pressure measurements were analyzed with an average of three measurements per patient. Of these measures, 46.5% were abnormal. The mean time from admission to documentation of the first blood pressure measurement was 2.5 minutes, with 42 minutes between subsequent measures. There is no correlation between the systolic blood pressure values and the mean time interval between blood pressure documentations: 0.173 (p=0.031).

**Conclusion:**

The present study found no correlation between frequency of blood pressure documentation and blood pressure values. The frequency of blood pressure documentation increased according to the severity of the patient and decreased during the length of stay in the emergency department.

## RESUMO

**Objetivo:**

Analisar a frequência de registros da pressão arterial realizados por profissionais de enfermagem em uma unidade de emergência.

**Métodos:**

Estudo transversal, observacional, descritivo e analítico, que incluiu registros de pacientes adultos admitidos em leitos de observação de uma unidade de emergência no período de março a maio de 2014. Os dados foram obtidos por meio de um instrumento de coleta de dados dividido em três partes: identificação do paciente, dados de triagem e registro da pressão arterial. Para a análise estatística, foi utilizado o coeficiente de correlação de Pearson, com nível de significância de α<0,05.

**Resultados:**

Foram analisados 157 prontuários e realizadas 430 medidas da pressão arterial, com média de três aferições por paciente; 46,5% dos valores obtidos estavam alterados. O tempo médio de admissão até o registro da primeira pressão arterial foi de 2,5 minutos, e de 42 minutos entre as medidas subsequentes. Não foi encontrada correlação entre os valores de pressão arterial sistólica e o intervalo médio de tempo entre os registros da pressão arterial: 0,173 (p=0,031).

**Conclusão:**

O presente estudo não encontrou correlação entre frequência de verificação da pressão arterial e os valores de pressão arterial. A frequência do registro da pressão arterial aumentou de acordo com a gravidade do paciente e diminuiu durante seu tempo de permanência no serviço de emergência.

## INTRODUCTION

Patients assessed in emergency departments (ED) who are at risk of clinical deterioration may be identified before a severe adverse event, by physiological changes observed by the nursing staff.^1^ A number of studies showed there is association between blood pressure (BP) abnormalities and the occurrence of adverse events, such as cardiac arrest, unplanned Intensive Care Unit admission, and increased mortality.^2-4^

Blood pressure documentation is made according to definition of care guidelines, physician orders and nursing clinical judgment, based on severity of the patient. Furthermore, the assessment of vital signs is frequently made by professionals who may not always understand the importance of this task, or have many different educational background or work experience.^5,6^

So far, the literature has no protocol available that determines the frequency of checking vital signs at the ED. Since 2007, the National Institute for Health and Clinical Excellence has published hospital clinical protocols for severely ill patients, and considers that monitoring of physiological parameters and early warning systems help healthcare professionals identify patients at risk of clinical deterioration.

These protocols also take into account that vital signs (heart rate, respiratory rate, systolic blood pressure, level of consciousness, oxygen saturation, and temperature) should be recorded at the initial assessment and, at least, every 12 hours, and that the frequency of monitoring should increase if changes in any physiological parameter are detected.^1^

The Emergency Severity Index (ESI) is an important triage tool, which provides patient’s risk classification according to their clinical condition and hospital resource.^7^ The ESI has been used in different countries and has shown a significant association between the categories of risk classification and the outcome of the patient at the ED.^8,9^ The identification of physiological changes during the ESI classification may predict the risk of clinical deterioration and the presence of severe illness.^10^

Although the BP measurement is one of the most frequently performed procedures in the clinical practice, the documentation and interpretation of its values as a decision-making tool is still not enough to ensure patient’s safety.^11,12^

## OBJECTIVE

This study aimed to analyze the frequency of blood pressure documentation performed by nursing professionals in an emergency department.

## METHODS

### Study design and setting

This is a cross-sectional, observational, descriptive and analytical study, conducted at the ED of a private hospital, located in the city of São Paulo, Brazil. In 2015, it was estimated that 130 thousand visits were made to this ED and 40 thousand patients were hospitalized by the specialties of Cardiology, Internal Medicine, Orthopedics, General Surgery, and Gynecology.

The ED observation wards were equipped with calibrated oscillometric BP devices, Infinity model, Delta series, manufactured by Dräger^®^ (Lübeck, Germany).

### Sample

The study included patient records of both sexes, aged over 18 years, who were admitted to the ED between March 5 and May 5, 2014. They were assessed by a nurse in triage and directed to an adult observation ward after evaluation by the clinician or Cardiologist. The sample calculation was not necessary, since all charts of patients admitted to the ED during the data collection were analyzed.

The charts of psychiatric patients, patients with emotional disorders or psychomotor agitation were excluded, which could interfere in BP measurement and documentation. A total of 162 patient charts were analyzed and five were excluded due to lack of required information.

### Ethical aspects

This study followed the national and international standards of ethics in research involving human subjects and the requirements of Resolution No. 466/2012. It was approved by the Research Ethics Committee of *Hospital Israelita Albert Einstein*, under protocol number 1.105.180/2015, CAAE: 31962014.1.0000.0071.

### Data collection

- Patient identification: the variables age, sex, time of admission, medical diagnosis/complaints, and medical resources used were analyzed.

- Triage data: ESI is a standardized risk classification based on the clinical conditions and on the need of hospital resources; it is made by a trained nurse, who assesses how long a patient can safely wait to be seen by a physician and treated. The ESI is a tool widely used in the United States and was validated by the Emergency Nurses Association and by the American College of Emergency Physicians. The physiological parameters were recorded by the nurse upon admission (systolic blood pressure, pulse rate, temperature, respiratory rate, and oxygen saturation), and used as basis to classify the patient into one of five possible levels: level 1 indicates the most urgent category, who require immediate care; level 2 classify patients in a high-risk situation; level 3 takes into account possible changes in physiological parameters of the patient and the need to use two or more resources in the ED to define the diagnosis; level 4 and 5 classify patients as not or as those who require only one or no additional resources in the ED.^7^

To facilitate data organization and interpretation, it was assumed that patients classified as levels 1 and 2 were in a high risk situation; patients classified in level 3 were in a stable condition; and patients classified in levels 4 and 5 were not urgent.

- Blood pressure documentation: it was defined as the number of BP measurements made by nursing staff during the patient’s stay at the ED. The BP measurements documented in the risk classification (triage) and during the length of stay of the patient in the observation wards were included.

Patients were considered as in unstable hemodynamic conditions if the systolic blood pressure was ≤90mmHg or ≥180mmHg.

### Data analysis

Descriptive statistics were used to examine the study variables and were expressed as means and standard deviation (SD). Data were entered twice into an Excel worksheet and exported to the Statistical Package for the Social Sciences (SPSS), version 20.0^®.^ To examine the relation between BP values and the frequency of BP measurements, Pearson’s correlation coefficient was used, with a significance level of α<0.05.

## RESULTS

A total of 157 patients were included in the study; in that, 83 (52.9) were female and 74 (47.1%) male. The mean age was 63 years (SD±20.8), range of 18 to 97 years. Most patients (96.2%) were assessed by a clinician, and 3.8% by a Cardiologist. Table 1 shows patient characteristics, BP values at triage, and presumptive diagnosis/complaints according to body systems.

**Table 1:** Patient characterization

Variables		
Age	63±21 years	
Sex n (%)		
Female	83 (52.9)	
Male	74 (47.1)	
Emergency department diagnosis or complaints n (%)		
Cardiovascular	13 (8.3)	
Neurological	16 (10.2)	
Respiratory	35 (22.2)	
Gastrointestinal	13 (8.3)	
General infectious	41 (26.1)	
Renal	19 (12.1)	
Others	20 (12.8)	

BP values at admission (Triage)	Mean	SD

SBP	131.1	26.4
DBP	74.8	15.5

BP: blood pressure; SBP: systolic blood pressure; DBP: diastolic blood pressure; SD: standard deviation.

The charts were classified according to the patient severity and the number of hospital resources used to define diagnosis; thus, 92 (58.6%) patients were in a high risk situation, 54 (34.4%) in a stable clinical condition, 2 (1.3%) in a less acute situation, and 9 (6%) charts did not have the triage level documented.

A total of 156 (99.4%) patients had their BP within normal parameters, however, when analyzing these values separately, 26 (16.6%) patients were hemodynamically unstable and 47 (29.9%) had a BP ≥140/90mmHg. In other words, 46.5% of patients had abnormal BP values.

The mean time from admission to first documentation of BP was 2.5 minutes, ranging from zero to 120 minutes.

A total of 430 BP measurements were performed, with a mean of three measurements per patient, ranging from one to eight in a period of 23 hours. All 157 patients had their BP measured upon admission and the number of BP measurements decreased according to length of stay.

During stay at the ED, an average of 2.9 BP measurements was performed in patients classified as at high risk, and 2.3 BP measurements in patients classified as being in a stable condition.

The BP was measured at an average of every 42 minutes, ranging from 15 minutes to 15 hours. The number of BP measurements for each patient varied from one to eight, and the longest interval occurred between the fourth and fifth measurements.

**Figure 1:**
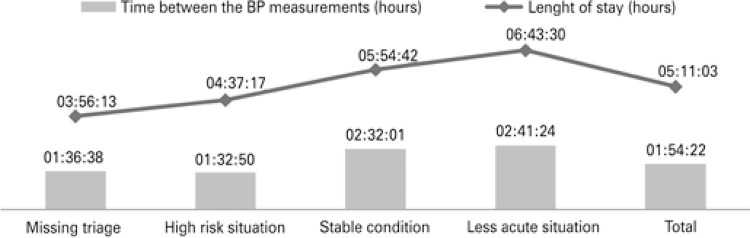
Mean time between the blood pressure measurements and mean length of stay at the emergency departments, according to patient severity level

Figure 1 show the average time between the BP measurements and the mean length of stay at the ED, according to patient severity level.

BP: blood pressure.

To examine the relation between BP values and the mean time interval between the BP measurements, a correlation analysis was performed. However, no correlation was found between systolic blood pressure and diastolic blood pressure values, considering the mean time interval between the BP measurements: r=0.173 (p=0.031), and r=0.116 (p=0.148), respectively.

## DISCUSSION

In the analysis of the BP documentation done by nursing professionals at the ED, there was no correlation between the systolic blood pressure/diastolic blood pressure values and the mean time interval between BP documentations.

In this study, most patients admitted to the observation ward were classified as in a high risk situation or stable condition, which meant that a large percentage of patients were at risk of quick clinical deterioration, and required a strict control of vital signs. While international studies^5,13^ showed that most patients admitted to the ED were classified as in a less acute situation, a recent study conducted in Brazil^14^ reported 63.5% of patients evaluated in the ED were classified as urgent or very urgent.

So far, there is a small number of studies on BP documentation at ED;^5^ in the same way, few studies have been done to identify patients at risk of clinical deterioration, and how vital signs can be used to develop protocols to support nursing professionals in their clinical practice.^15,16^ The results of this study may contribute to recognizing nursing knowledge gaps on BP documentation and ensure safety of patient admitted to the ED.

Unlike results reported in this article, a study carried out in the United States, in 2011, demonstrated there is a significant difference in the frequency of documenting BP measurements and the risk classification of the ESI. Patients classified as in a stable condition had the longest interval between BP measurements (167.5 minutes), if compared to those in a less acute situation (89.2 minutes).^5^

Moreover, the patients classified at high risk had their BP measured at intervals of 1 hour and 32 minutes, in average; while patients classified as in stable conditions and less acute situations had it measure at every, in average, 2 hours and 32 minutes, and 2 hours and 41 minutes, respectively. This fact can be associated with the patient risk of clinical deterioration, nursing judgment, and BP management in the ED.

The differences between the frequencies of BP measurement at the ED suggest the need to implement standardized protocols to ensure knowledge of nursing professionals about the interpretation of vital signs. The reasons for this difference were beyond the scope of this project and deserve further investigation on the topic.

Recognizing changes in BP values, as well as the frequent BP measurement, are essential care activities for severe patients to assess treatment effects, detect procedural complications, and prevent severe adverse events, such as cardiopulmonary arrest, unplanned admission to intensive care unit and increased early mortality.^2^In this study, the mean BP values were within the normalcy parameters during the risk classification, but presented changes during the length of stay on the observation wards. Other studies conducted at an ED had similar results, and related these values to a wide variety of complaints, diagnoses, health conditions, and hospital management processes upon admission.^17-19^

Although vital sign monitoring is one of the most commonly performed tasks at an ED, there is limited information regarding the optimal frequency to BP measurement. In general, BP measurement depends on hospital policy, nursing clinical judgment, physician’s orders, presumptive diagnosis and symptoms of the patient.^20^

The literature shows that lack of accuracy in BP measurement, recording, and interpretation can be justified by excessive workload, difficulty in interpreting and recognizing changes in vital signs, and difficulties to make decisions about severe patients care.^21^

Some limitations of this study should be considered: inclusion of a small number of charts without randomized control, which limited the range of results; the study site did not allow comparison with other hospitals, since it is a private institution, with specific infrastructure and technological management system, which are superior to those of other healthcare organizations in Brazil; the investigator could not maintain neutral relation for being a nursing staff, and directly involved with medical records assessed; the indirect BP measurement was not evaluated regarding the stages validated by the 6^th^ Brazilian Hypertension Guidelines.^22^

## CONCLUSION

The present study found no correlation between the systolic blood pressure or diastolic blood pressure values and the mean time interval between blood pressure documentations. Besides the frequency of blood pressure documentation increased according to patient severity and decreased during the patient’s length of stay at emergency departments. Considering the large number of patients with abnormal blood pressure values and the shortage of decision-making tools for nurses to manage these vital signs, it is suggested the development of blood pressure monitoring guidelines that can ensure the safety of patients admitted to emergency departments.
